# CANDy: Automated analysis of domain architectures in carbohydrate-active enzymes

**DOI:** 10.1371/journal.pone.0306410

**Published:** 2024-07-11

**Authors:** Alex Windels, Jorick Franceus, Jürgen Pleiss, Tom Desmet

**Affiliations:** 1 Department of Biotechnology, Centre for Synthetic Biology (CSB), Ghent University, Ghent, Belgium; 2 Institute of Biochemistry and Technical Biochemistry, University of Stuttgart, Stuttgart, Germany; INRAE, FRANCE

## Abstract

Carbohydrate-active enzymes (CAZymes) can be found in all domains of life and play a crucial role in metabolic and physiological processes. CAZymes often possess a modular structure, comprising not only catalytic domains but also associated domains such as carbohydrate-binding modules (CBMs) and linker domains. By exploring the modular diversity of CAZy families, catalysts with novel properties can be discovered and further insight in their biological functions and evolutionary relationships can be obtained. Here we present the carbohydrate-active enzyme domain analysis tool (CANDy), an assembly of several novel scripts, tools and databases that allows users to analyze the domain architecture of all protein sequences in a given CAZy family. CANDy’s usability is shown on glycoside hydrolase family 48, a small yet underexplored family containing multi-domain enzymes. Our analysis reveals the existence of 35 distinct domain assemblies, including eight known architectures, with the remaining assemblies awaiting characterization. Moreover, we substantiate the occurrence of horizontal gene transfer from prokaryotes to insect orthologs and provide evidence for the subsequent removal of auxiliary domains, likely through a gene fission event. CANDy is available at https://github.com/PyEED/CANDy.

## 1. Introduction

Continuous advances in high-throughput (meta)genomic technologies and decreasing sequencing costs have led to the exponential growth of available sequence data. Associated with this expansion is the need for sequence analysis methods and classification systems, allowing researchers to efficiently navigate through this vast amount of information. Nowadays, numerous predictive tools are available for the automated annotation of functional motifs, protein domains, and structural folds in protein sequences based on experimental data of characterized homologs [1–6]. The objective of these *in silico* tools is to streamline the process of selecting proteins that may be of interest for further experimental work or computational studies and helping researchers make informed decisions on which candidate proteins to prioritize. This step is crucial since only a fraction of the enormous collection of proteins can ever be studied *in vitro* [7].

A highly regarded example of such a tool is InterPro. InterPro is a consortium of several protein signature databases such as Pfam, PRINTS, PROSITE, SMART and additional resources, including MobiDB-lite, SignalP, TMHMM and AntiFam [4]. By combining the complementary expertise of each of those member databases, InterPro facilitates functional analysis of proteins through the prediction of domains and conserved sites and the classification of proteins into families. This classification provides valuable insights into the function of proteins, evolutionary relationships and interactions within biological systems. In recent decades, there has been a noticeable surge in the number of databases dedicated to collecting and organizing functionally related proteins, resulting in curated collections like the plastics-active enzymes database (PAZy), phage lytic proteins database (PhaLP) and carbohydrate-active enzyme database (CAZy) [8–10].

The latter database (www.cazy.org) became publicly available in 1998 and has since played a vital role in enabling the analysis of the capabilities of organisms in utilizing, assembling, and degrading carbohydrates. Their sequence-based classification approach of carbohydrate-active enzymes allows for a swift discovery and annotation of carbohydrate-active enzymes, covering five distinct protein classes i.e., glycoside hydrolases (GH), glycoside transferases (GT), polysaccharide lyases (PL), carbohydrate esterases (CE) and auxiliary activities (AA). The CAZy database continues to experience rapid growth. The number of included sequences has increased 6.6-fold between 2013 (344,698) and 2021 (2,270,027) [10]. This growth leads to the ongoing expansion of established protein families and the frequent identification of numerous novel families, yet it also results in a loss of a finer level of granularity and specificity. Consequently, CAZy families are regularly subdivided into subfamilies, thereby facilitating a deeper comprehension of sequence-function correlations and evolutionary relationships [11–13].

Understanding the phylogenetic context of a carbohydrate-active enzyme within its family or subfamily is undoubtedly crucial. However, it is equally important to consider the enzyme’s domain composition. Carbohydrate-active enzymes often possess multiple catalytic domains, as well as other functional modules such as carbohydrate-binding modules (CBMs) and linker domains [14–16]. The domain composition of an enzyme plays a significant role in determining its overall function and substrate specificity [17]. Each catalytic domain may target different carbohydrate substrates or exhibit distinct catalytic activities, while additional modules such as CBMs can enhance the efficiency of the enzyme by facilitating substrate recognition and binding [18]. Furthermore, studying the domain architecture of carbohydrate-active enzymes can improve our understanding of their evolutionary history and the mechanisms underlying their diversification [19]. Novel or adapted enzymatic functions may emerge through domain rearrangements, fusion events, or the acquisition or loss of additional domains [20].

Here we introduce the Carbohydrate Active eNzyme Domain analysis tool (CANDy), an open-source pipeline developed to meticulously analyze the domain composition and architecture of all members in a CAZy family of choice. CANDy’s framework combines a collection of novel scripts with existing tools, packages, and databases, enabling a rapid and seamless identification of protein domains across any CAZy family. The primary objectives of CANDy encompass three key aspects: (I) facilitating the exploration and discovery of novel multi-domain enzymes, (II) assisting in the phylogenetic analysis through the integration of phylogenetic, biochemical, and domain architectural data, and (III) supporting the construction of tailored CAZymes comprising specific protein domains, by providing a comprehensive overview of existing domain architectures, their prevalence, phylogenetic distribution and the frequency of distinct domain types along with their interconnections.

## 2. Materials and methods

### 2.1. CANDy architecture

**Fig 1:**
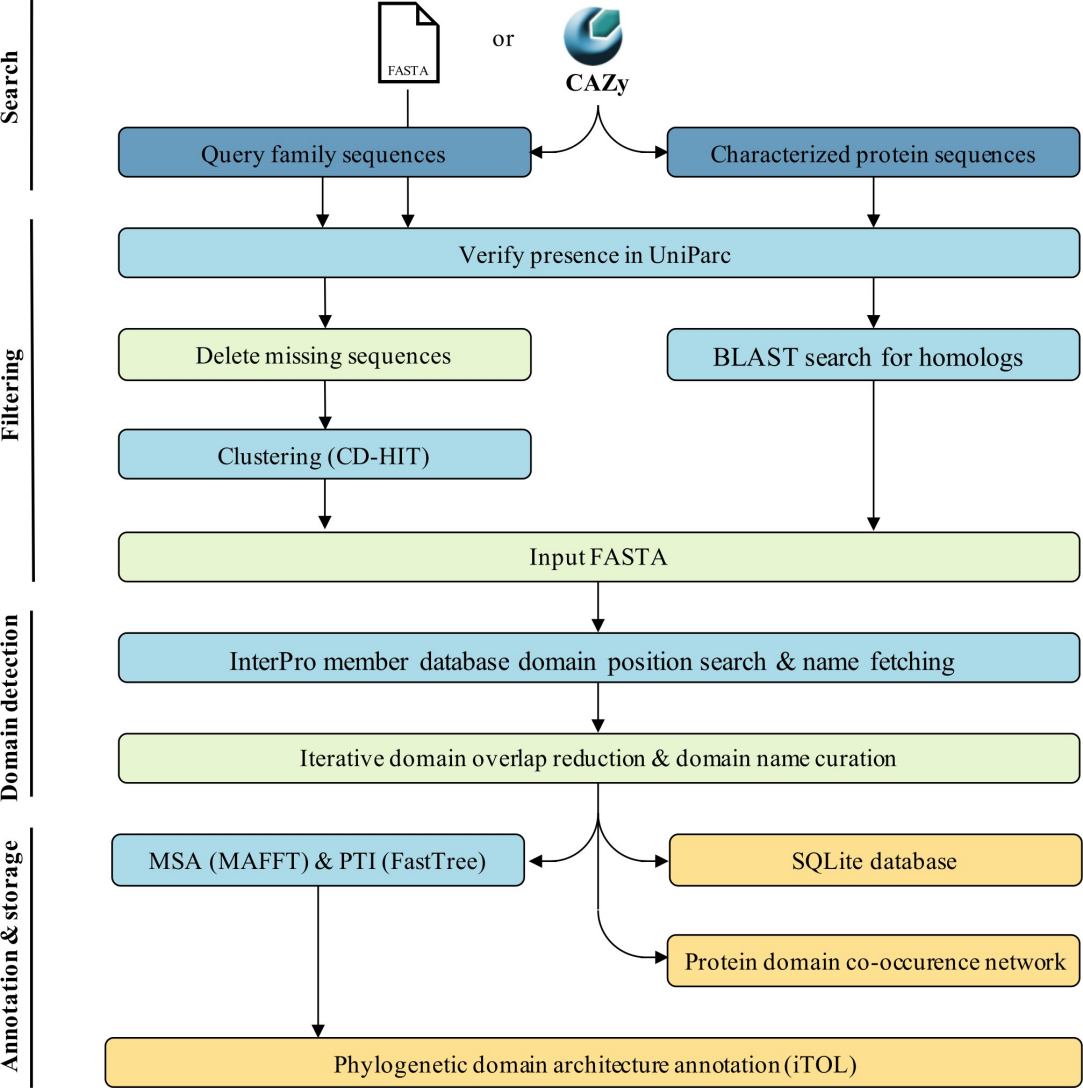
The CANDy workflow. The workflow consists of four distinct steps: (I) Searching and fetching all protein sequences included in the query CAZy (sub)family, (II) filtering of unique sequences present in the UniParc database, (III) search for protein domain information and iterative clean-up of the results and (IV) database construction and protein domain annotation. CANDy only requires the CAZy (sub)family of interest as input and offers additional options for more specialized queries.

CANDy implements a four-step workflow: (I) Searching and fetching the identifiers and protein sequences that belong to the query family, (II) filtering the input sequences, (III) searching protein domains and (IV) annotating the identified domains (Fig 1). The user has the option to input any existing CAZy family or subfamily, to specify a taxonomic subset of interest (All, Archaea, Bacteria, Viruses, Eukaryota, or Unclassified i.e., proteins from uncultured or synthetic organisms) and to proceed with the construction and annotation of a phylogenetic tree. A more selective query can reduce calculation times and save computational resources. Alternatively, users have the option to upload a FASTA file containing a custom set of sequences, which are not required to be related to CAZymes, as the workflow is designed to accommodate all types of proteins.

In the sequence search and retrieval step, the GenBank identifiers of the proteins associated with the queried (sub)family are retrieved from CAZy. Subsequently, the NCBI API (https://www.ncbi.nlm.nih.gov/) is utilized to retrieve the protein sequences corresponding to each identifier, at a rate of 3 requests/second. In cases where users choose to input a FASTA file containing query sequences, this step is bypassed, and the workflow advances to the subsequent stage.

In the subsequent sequence filtering step, the UniProt API (https://www.uniprot.org/) is employed to retain sequences that are available in UniParc. The UniParc database stands as the most comprehensive and non-redundant repository, housing all protein sequences sourced from major publicly accessible protein sequence databases [21]. Any sequences that are absent from UniParc are excluded from the workflow due to the absence of domain or site information for those proteins in the InterPro database (https://www.ebi.ac.uk/interpro/). Since InterPro provides annotations for all UniParc proteins, it stands as the most extensive source of information on protein domains and site and it provides annotations for nearly all CAZy proteins.

Finally, a clustering step (CD-HIT) is performed to reduce the number of input sequences [22]. Given our interest in the domain architecture of the queried family, the clustering cut-off can be set relatively low (default = 85%). Users can also specify the minimum alignment coverage for both the shorter and longer sequence, which defaults to 0%. Notably, the workflow also fetches the list of experimentally characterized proteins from CAZy, together with their EC number and includes their sequences in a FASTA file, even if they would have been eliminated during the clustering step. In the rare case a characterized protein is not found in UniParc, a protein BLAST search is conducted. The sequence of the highest BLAST result will be included for the characterized protein instead, provided that the sequence similarity–calculated as the ratio between the number of identical matches and the query protein length–is at least 95% (default). For efficiency reasons, a BLAST search is not performed on missing non-characterized proteins, as close homologs are likely already present in the dataset and the BLAST result may be removed during the clustering step.

The domain detection step is the most critical phase of the workflow and involves identifying protein domains within the query sequences. To accomplish this, the InterPro API is leveraged, which provides comprehensive results for all UniParc proteins. The domains are sourced from a range of databases, including CATH-Gene3D, CDD, HAMAP, MobiDB Lite, PANTHER, Pfam, PIRSF, PRINTS, PROSITE profiles, SFLD, SMART, SUPERFAMILY, and NCBIFAMs [6, 23–34]. Next, the InterPro API is used once again to retrieve the corresponding domain names corresponding to the domain identifiers obtained through InterPro.

Since the CANDy pipeline accesses multiple databases, multiple hits may be detected for a single protein region, each carrying a distinct name. To ensure that each region is annotated by no more than one domain name, an iterative cleaning step is performed. During this step, adjacent domains are searched for significant overlaps. Two regions are considered to encode the same domain if the overlap exceeds a certain threshold, which is set at 31% be default. This threshold was determined by analyzing ∼200,000 domain annotations across ∼22,000 proteins, revealing an average overlap of 31% among retrieved annotations. However, users can adjust the stringency of this threshold as needed.

At most one of the overlapping domains will be retained, provided the following conditions are met: (a) the domain has a valid name and is not annotated as ‘None’; (b) the domain does not encode a signal peptide, identified by annotations containing ‘SIGNAL’ or retrieved from the PHOBIUS database [35]; and (c) the domain size lies within a given range, set by default between 10 and 800 amino acids. The domain size condition was designed to filter out annotations that are irrelevant to CANDy, such as annotations of conserved sites or annotations that cover multiple domains. The default upper limit was chosen after an analysis of ∼200 InterPro accessions with ‘Glycosyl’ in their names across ∼26,000 proteins, where the largest domain size was found to be 621 AA. If both overlapping domains comply to all three rules, one domain is chosen based on database preference, with options to adjust database priority as needed. Additionally, since identical domains can be known by different names across databases, users can engage in a straightforward domain name curation process. During this step, users can designate (custom) overarching names to replace a few of the automatically detected names.

In the fourth and final domain annotation and data storage step, all sequences, origin details, and domain architectures generated by CANDy are stored in an SQLite database. Leveraging the sqlite3 library, the architecture of the SQLite database was designed with three key objects or tables. The ’ProteinSequences’ table stores comprehensive information regarding the query sequences and their associated domain architectures. The ’DomainAssemblies’ table summarizes all the unique combinations of domains identified within this family. Lastly, the ’DomainCuration’ table retains records of any changes made to domain names during the curation process, ensuring accurate tracking of modifications.

The results of CANDy’s protein domain analysis can optionally be visualized in two ways. First, a protein domain co-occurrence network is generated, which offers a rapid visualization on the frequency of domain occurrences and their interconnection. Second, users have the option to generate a phylogenetic tree, annotated with the domain architectures. Therefore a multiple sequence alignment (MSA) using MAFFT with default settings is performed [36]. Proteins that do not contain any detected domains are excluded from the alignment. Subsequently, phylogenetic tree inference is carried out using FastTree with default settings and the resulting tree is outputted in Newick format [37]. Local support values for each split are estimated using the Shimodaira-Hasegawa test and are based on 1000 resamples (default). Finally, two iTOL annotation files are generated [38]. The first file allows for the annotation of leaf nodes with the respective protein’s domain architecture, where each domain is represented by a unique shape and color. The second file assigns distinct colors to the labels of characterized sequences, highlighting each specificity. In the case an enzyme possesses multiple activities, a warning message when running CANDy pops up, indicating that the protein has multiple activities but only one will be used to annotate the phylogenetic tree For Jupyter Notebook users, the annotated tree can be visualized using the ete3 toolkit [39].

It is important to clarify that CANDy does not conduct protein domain predictions on its own; rather, it leverages the outcomes of other predictive models. As a consequence, the effectiveness and comprehensiveness of CANDy’s outcomes are contingent upon the quality and scope of annotations available within these databases.

### 2.2. CANDy analysis of family GH48

The GH48 family was chosen to illustrate CANDy’s functionalities. Initially, 1712 identifiers were extracted from CAZy, with 1651 of these sequences present in UniParc. Duplicate identifiers or sequences and partial proteins were discarded, and the remaining sequences were clustered at an 85% similarity threshold. A total of 216 distinct sequences were retained, including 18 characterized proteins. CANDy identified and annotated the domains, after which the automatically retrieved domain names were customized using the built-in domain name curation process (S1 Table). Setting minimal alignment coverage values to 90% for more stringent clustering did not affect the diversity of detected domains. The protein sequences were then incorporated in a custom BLAST database. In the few cases where two separate UniParc member databases only partially detected the catalytic domain in sequences, the domain edges were automatically adjusted using the outermost positional values. Next, 670 unique GH48 sequences underwent BLAST analysis against this database. This approach facilitated the estimation of the potential inclusion of GH48 sequences within each unique domain arrangement. Finally, the domain co-occurrence network was visualized in Cytoscape using the yFiles Organic Layout [40].

### 2.3. Protein domain evolution analysis

Horizontal gene transfer events within the GH48 family were investigated by comparing protein domain homology between two distinct groups of Actinomycetote proteins, consisting of 36 and 28 sequences, respectively. To conduct this analysis, the sequences of the catalytic domain and, when present, the CBM and immunoglobulin-like domain from each protein sequence were extracted, using the domain boundary information sourced from the SQLite database. Subsequently, pairwise alignments were performed between these domain types using the Bio.pairwise2 algorithm. The percentage identity between sequences was computed by dividing the best alignment score by the highest length of the two query sequences. Next, statistical analysis was conducted to compare the mean identity of each of these groups, involving the utilization of the Kruskal-Wallis test, followed by a Dunn’s post hoc analysis.

## 3. Results and discussion

### 3.1. CANDy pipeline

**Fig 2:**
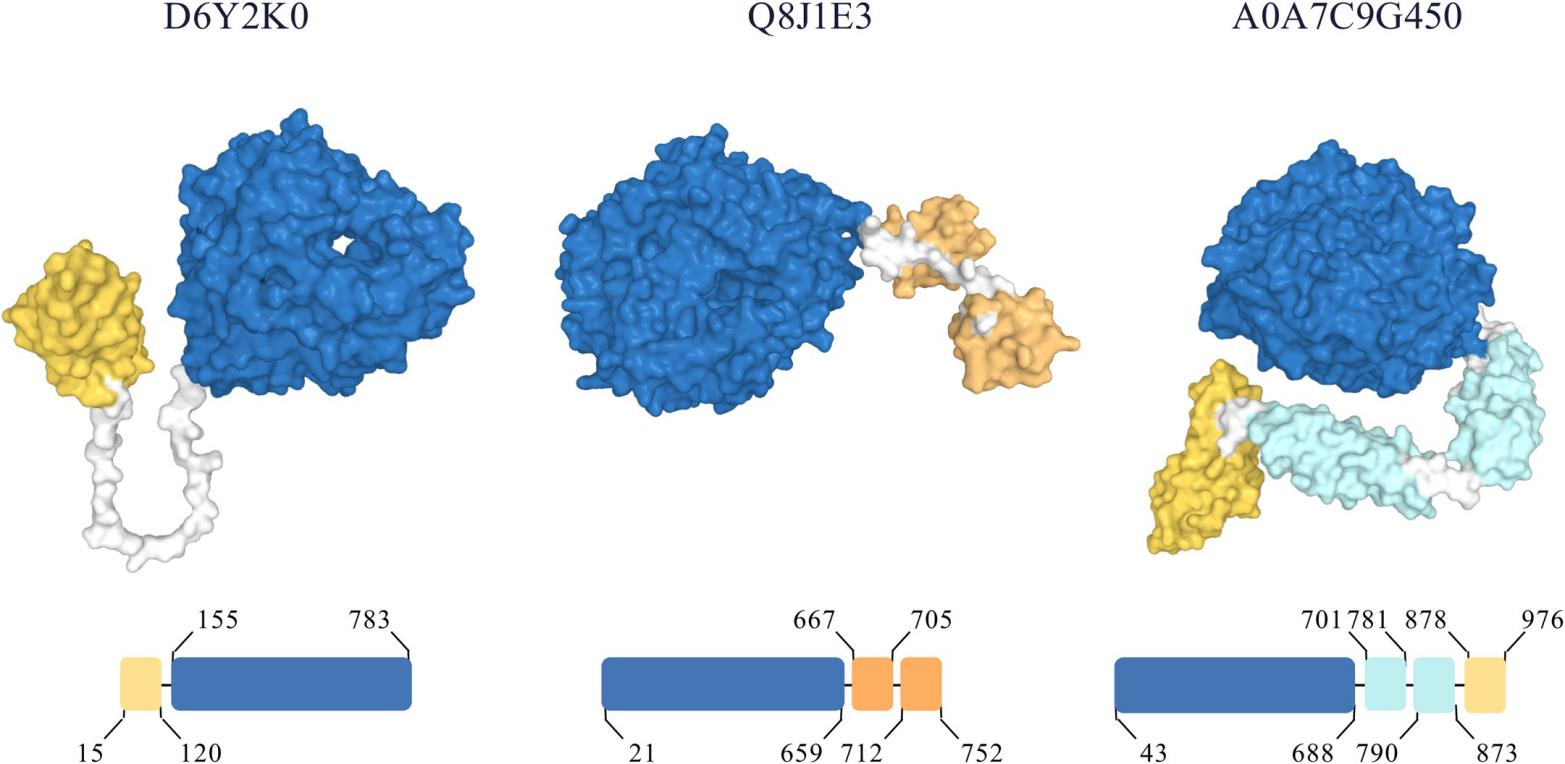
Example of CANDy’s protein domain annotations for three GH48 proteins. UniProt identifiers are displayed above the AlphaFold protein structures, alongside a simplified representation of the domain boundaries. Shown proteins domains are CBM’s: yellow; catalytic domains: blue; dockerins: orange and immunoglobulin-like domains: cyan. Linker regions are in white.

CANDy’s workflow comprises four stages (Fig 1). First, the system retrieves identifiers for all proteins within the query (sub)family from CAZy and subsequently fetches their corresponding amino acid sequences. These sequences serve as the basis for the entire analysis. In the second step, the collection of input sequences is cleaned through the deletion of protein sequences for which no InterPro annotations exist. A sequence homology reduction step is executed to reduce the size of the input file and increase the speed of the subsequent analysis, while retaining all sequences of characterized proteins. In the third phase, an InterPro search is performed for each sequence, retrieving domain annotations from its member databases. This process yields multiple (potentially redundant) annotations per protein. To consolidate a concise domain composition output, an iterative annotation cleaning step is implemented, resulting in a complete and precise domain annotation (Fig 2). Finally, the results are stored in an SQLite database to simplify database sharing and distribution. To delve deeper into the results of CANDy a protein domain co-occurrence network is generated. Also, users have the option to perform a phylogenetic tree inference (PTI) step. This facilitates the visualization of evolutionary relationships among proteins in the query family. Furthermore, the obtained tree can be enriched with domain architecture annotations at the leaf nodes so that the presence of specific domains and their loss or acquisition between clades can be observed at a glance.

CANDy is an open-source pipeline that is conveniently available on Google Colab (https://colab.research.google.com/drive/1pxUSkl6Bv2JfAZ4-3ljsEdWlocqm_RXB?usp=sharing), eliminating the need for any installation requirements. However, it is important to note that the free version of Google Colab has limited computational resources, making it suitable primarily for smaller (or selections of) CAZy families. When analyzing larger families or when improved performance is desired, the pipeline can also be run locally using the Jupyter Notebook version of CANDy, available for download on GitHub (https://github.com/PyEED/CANDy).

### 3.2. CANDy analysis of family GH48

CANDy was employed for glycoside hydrolase family 48 (GH48), a small yet diverse family that is currently known to contain four specificities: reducing end-acting cellobiohydrolases (EC 3.2.1.176), endo-β-1,4-glucanases (EC 3.2.1.4), chitinases (EC 3.2.1.14), and cellobiohydrolases (EC 3.2.1.91). The family comprises 18 characterized proteins, of which 11 crystal structures have been solved. Our analysis revealed that all of the characterized proteins cover eight different domain assemblies, of which all domains can be classified into four distinct types based on their sequence and structure: the GH48 catalytic domain (which hereafter will be named ‘catalytic domain’), CBM domains, dockerin domains, and immunoglobulin-like domains (Table 1).

**Table 1:** Predicted protein domain architectures of characterized GH48 proteins. The observed prevalence indicates the fraction of characterized proteins that can be included in the specific domain architecture.

Predicted domain architecture	Observed prevalence (%)	Taxonomy	Example protein (GenBank ID)	Known specificities
Catalytic domain	23	Bacteria, Eukaryotes	AAU40776.1	3.2.1.4,
				3.2.1.14,
				3.2.1.91, 3.2.1.176
Catalytic domain + CBM	4.5	Bacteria	ABN51312.1	3.2.1.4
Catalytic domain + Dockerin	3.6	Bacteria, Unknown	AEV70443.1	3.2.1.4, 3.2.1.176
Catalytic domain + Immunoglobulin-like + CBM	10	Bacteria	ABX43721.1	3.2.1.176
CBM + Immunoglobulin-like + Catalytic domain	43	Bacteria	AAD39947.1	3.2.1.176
Catalytic domain + Immunoglobulin-like + Immunoglobulin-like + CBM	2.7	Bacteria	ABC29272.1	3.2.1.4, 3.2.1.176
Catalytic domain + Immunoglobulin-like + Immunoglobulin-like + Immunoglobulin-like + CBM	1.9	Bacteria	AAB00822.1	3.2.1.176
Catalytic domain + CBM + CBM + CBM + Catalytic domain	1.0	Bacteria	ACM60955.1	3.2.1.176

**Fig 3:**
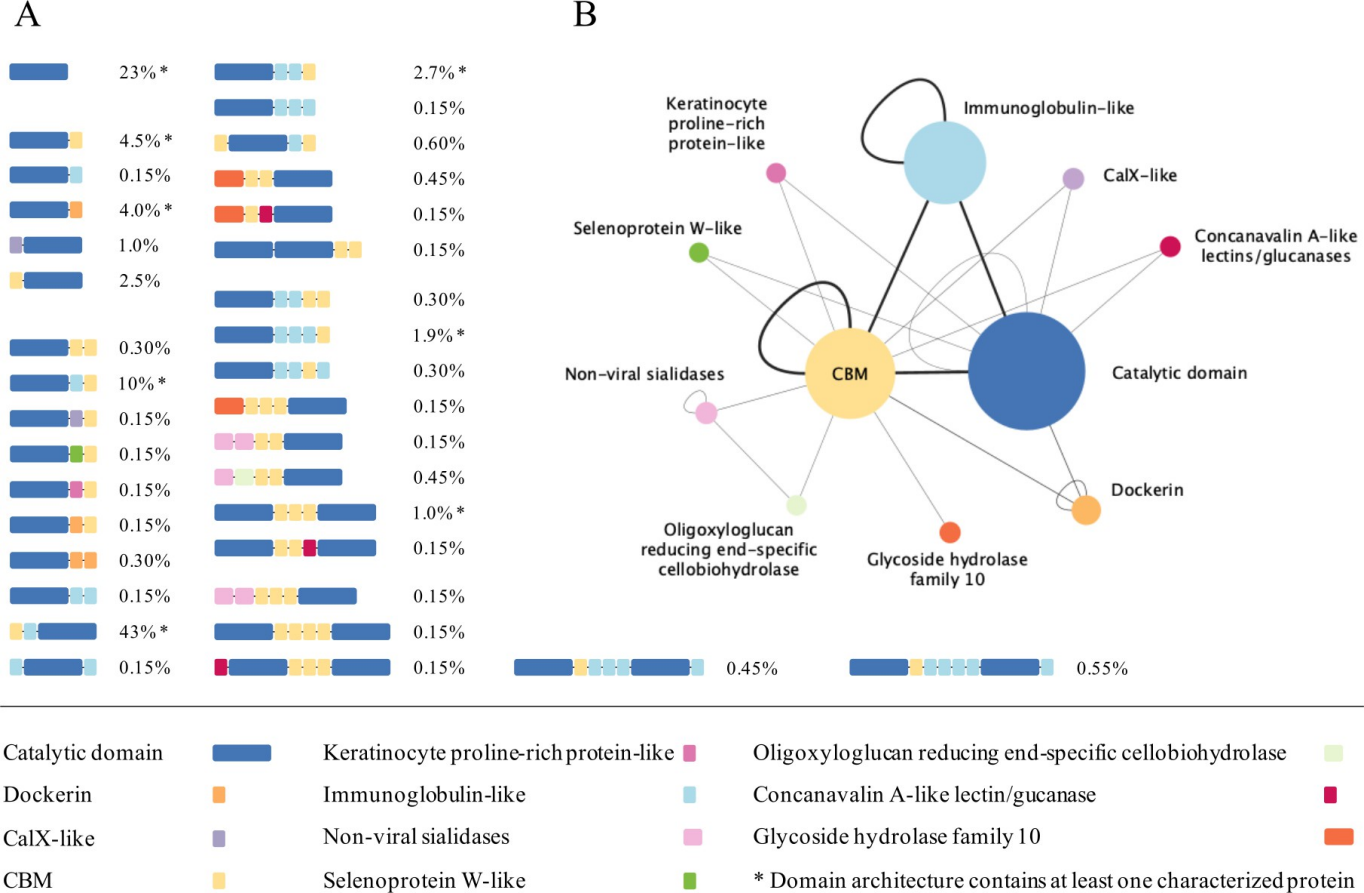
A. Overview of the 35 predicted domain architectures in family GH48. Domain architectures containing at least one characterized protein are marked with an asterisk. The sizes of domains are proportional to the average size of catalytic domains. B. Protein domain co-occurrence network as output from CANDy. The size of each node reflects the occurrence of that domains in family GH48 (min: 1, max: 225). The width of the edges corresponds to the frequency of connections between identical types (loops) or diverse types of domains (min: 1, max: 163); thicker edges denote frequent adjacency between respective domains.

A custom BLAST analysis conducted on all unique sequences from this family showed that 90% of the GH48 sequences can be attributed to one of these eight architectural categories. Among them, the most common architecture is the combination of a CBM with an immunoglobulin-like domain and a catalytic domain, in that order (47% of all sequences), followed by proteins containing only a catalytic domain (23% of all sequences). However, the remaining ten percent of proteins sequences show 27 different domain architectures that are not found in any of the characterized representatives (Fig 3A).

The identified domain architectures exhibit significant diversity, showing variations not only in their composition but also in the number of interconnected domains. According to our analysis, GH48 proteins can adopt various domain configurations, ranging from a single catalytic domain to assemblies of up to eight modules. Notably, there are 17 sequences that display a unique domain architecture, rendering them interesting candidates for future research and comprehensive experimental characterization (S2 Table).

Next to the discovery of novel architectures, CANDy is also able to pinpoint sequences that contain atypical types of domains as well. The results show that a number of GH48 enzymes may exhibit additional activities due to the presence of other catalytic domains such as those annotated as sialidases, GH10 domains, concanavalin A-like lectins/glucanases and oligoxyloglucan reducing end-specific cellobiohydrolases. Moreover, six architectures appear to contain two GH48 catalytic domains. Only the concanavalin A-like lectins/glucanase module is found to be directly linked to GH48 catalytic domains, while the other catalytic domains are separated by at least a CBM or another type of catalytic domain (e.g., ‘non-viral sialidase + oligoxyloglucan reducing end-specific cellobiohydrolase + CBM + CBM + GH48 catalytic domain). CANDy thus enables the rapid and high-throughput detection of these unexpected domains and potential new or additional activities in a family containing several hundred or thousands of protein sequences, a task which was previously unfeasible.

Furthermore, CANDy offers users a co-occurrence network that visually represents both the frequency of different domain types and the degree to which they are interconnected (Fig 3B). The GH48 catalytic domain is obviously the most prevalent domain in this family, constituting 39% of all identified modules. It is followed by CBMs (28%), immunoglobulin-like domains (25%), dockerins (4%), non-viral sialidases (1%), and all other modules, each representing less than 1% of the detected domains. Interestingly, despite their dominance in the domain landscape, catalytic domains rarely appear twice within a single protein sequence, accounting for only 2.8% of occurrences, and they are only once found to be interconnected. Several CBMs and/or immunoglobulin-like domains usually tend to serve as intermediary linkers.

### 3.3. Evolution of domain architectures

**Fig 4:**
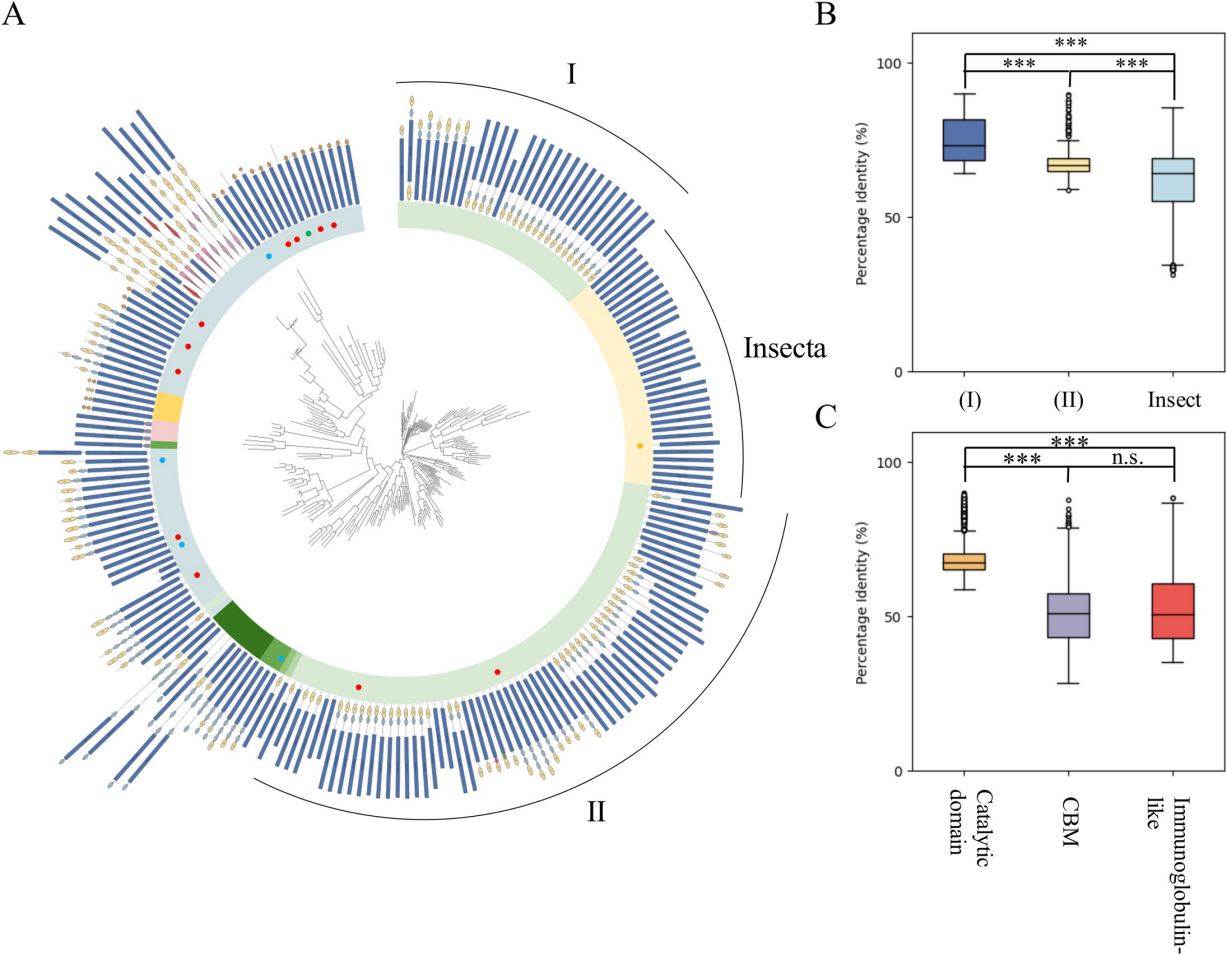
Phylogenetic tree as output from CANDy. The leaf labels are annotated with their respective domain architecture, for which the same color code is used as in Fig 3. Taxonomic groups are distinguished by various hues, including a gradient from light to dark green for Actinomycetota, Mycococcota, Chloroflexota, Pseudomonadota, and Bacteroidota, light blue for Bacillota, light yellow for Insecta, dark yellow for Fungi, and pink for viruses. Characterized proteins are annotated with a dot, blue: EC 3.2.1.14; yellow: EC 3.2.1.14; green: EC 3.2.1.91; red: EC 3.2.1.176. The two groups of Actinomycetota proteins are marked with I and II. B. Boxplot of the percent identity between the catalytic domains of the two groups of Actinomycetota (dark blue and yellow) and Insecta (light blue). C. Boxplot of the percent identity between the catalytic domains (yellow), CBMs (violet) immunoglobulin-like domains (red) of all Actinomycetota sequences. ^***^: p < 0.001; n.s.: not significant.

In addition to examining relationships at the protein domain level, CANDy also generates a phylogenetic tree, enabling the exploration of the evolutionary relationships among the proteins of interest. The leaf labels of the characterized proteins are automatically colored by substrate specificity, facilitating functional analysis and selection of promising candidates for experimental characterization. Furthermore, all proteins included in the phylogenetic tree are annotated with their respective domain architecture (Fig 4A) (S1 Fig). This visualization provides a comprehensive and insightful overview of the prevalence and distribution of domain architectures and their constituent protein domains.

Family GH48 currently covers enzymes from viruses, six prokaryotic phyla (Actinomycetota, Mycoccotota, Chloroflexota, Pseudomonadota, Bacteroidota and Bacillota) and two eukaryotic kingdoms (Fungi and Animalia). Given the position of the latter two clades within the Actinomycetota and Bacillota clades, respectively, it has been hypothesized that the eukaryotic GH48 sequences originated from a horizontal gene transfer (HGT) event. Our tool allows such events to be studied in more detail. For example, when focusing on the orthologous insect sequences, situated between the two groups (I and II) that make up the Actinomycetota clade, the differences in domain architecture make it clear other fusion and/or fission events occurred before or after the HGT event (S2 Fig). Leveraging the domain positions provided by CANDy, the identified domains can easily be excised from the complete protein sequences, to study sequence identity and divergence at the level of an individual domain. The similarity of the catalytic domains within the two groups Actinomycetote sequences (I: 74.7% and II: 68.3% respectively) is significantly higher than the identity between insect catalytic domains (61.5%) (Fig 4B) (S3 Table). This discrepancy can be explained by the tendency of animal mutation rates to be higher than prokaryote mutation rates, suggesting that HGT occurred early in the evolutionary timeline [41–44]. The common ancestor of this event likely contained a catalytic domain and at least one CBM and immunoglobulin-like domain. Subsequently, during eukaryotic evolution, gene fission may have occurred, leading to the removal of the auxiliary domains from the protein.

Further analysis reveals that the overall homology of Actinomycetota CBMs and immunoglobulin-like domains are 51.3% and 53.4% respectively, which is significantly lower than the homology between the catalytic domains (69.1%) (Fig 4C) (S4 Table). This finding, alongside the extensive architectural diversity of the GH48 family, supports the previously formulated hypothesis that functional innovation in GH48 paralogs may predominantly arise from the auxiliary domains rather than the catalytic domain [42]. While previous studies have shown the significance of CBMs in the adsorption and kinetics of GH6 and GH7 cellobiohydrolases, as well as the role of fibronectin 3 (immunoglobulin-like) domains in surface modification and cellulose hydrolysis by GH9 cellobiohydrolases, these effects remain unexplored in family GH48 enzymes [45–47].

### 3.4. CANDy: A collaborative project that expands the toolbox

By making CANDy accessible on GitHub and Google Colab, the scientific community can utilize the workflow for their specific research requirements and projects. By making CANDy accessible on GitHub and Google Colab, the workflow can be used freely, and it can be adapted, revised or expanded through collective community efforts. Therefore, CANDy promotes FAIR data principles and facilitates the reproducibility of research outcomes. Although CANDy was specifically developed for the analysis of domains in CAZymes, the pipeline can easily be run on any set of protein sequences when the user provides a custom FASTA file as input. This adaptability opens the possibility of extending enzyme discovery and engineering tools like EnzymeMiner to expand their analysis on a domain level [48].

CANDy presents a complementary addition to the collection of existing CAZy sequence analysis tools. CAZy_webscraper was previously developed for automating data retrieval from the CAZy database, while dbCAN and CUPP allow researchers to automatically annotate CAZymes within genomic or other input sequences [49–51]. This is done by a sequence-based classification approach, either by establishing signature domains for each CAZyme family using HMMs or utilizing an unsupervised peptide-based clustering algorithm. Although both tools provide domain information for CAZymes, their annotations are constrained to domains contained within their respective training datasets, namely catalytic domains and CBMs. Consequently, auxiliary domains such as linker domains, non-carbohydrate active catalytic domains, and modules involved in the assembly of large multienzyme complexes like cellulosomes are omitted from the results. With CANDy annotation data now can be sourced from 13 specialized databases, which allows a broader spectrum of domains to be included in the analysis, beyond just those that are directly associated with carbohydrate binding or conversion. As these databases continue to expand and model quality improves, CANDy’s annotation capability is assured to become more complete and precise over time. CANDy distinguishes itself from previously reported tools by not only automatically annotating protein domains for an entire CAZy family, but also by automatically providing a visual overview of the results in a phylogenetic tree or protein domain co-occurrence network. This underscores CANDy’s primary objective of enabling users to conveniently conduct a thorough all-in-one domain architecture analysis for any CAZy family or custom sequence dataset.

Compared to doMosaics, another software for protein domain detection and visualization, CANDy offers several advantages [52]. Firstly, CANDy is completely open-source, allowing continuous adaptation, updates, and improvements. Secondly, the Google Colab version of CANDy requires no installations, making it accessible on any internet-connected device. Alternatively, SACCHARIS offers a pipeline tailored to the identification of uncharacterized sequences for the exploration of new CAZyme or CBM specificities [53]. Unlike SACCHARIS, which lacks information on protein domain assemblies and their distribution across the tree, The results of CANDy can be valuable for candidate selection, particularly in families that contain few characterized proteins. Moreover, CANDy compiles all analyzed sequences, metadata, and domain architectures into an SQLite database, facilitating integration with other tools and enhancing its utility within scientific workflows. Finally, CANDy is highly complementary to FAS, a tool that excels in assessing the similarity of protein feature architectures by calculating a measure of the similarity between the feature architectures of proteins. This aids various bioinformatics analyses. Finally, CANDy is highly complementary to FAS, a specialized tool that excels in evaluating the similarity of protein feature architectures by comparing domain structures and other protein features [54]. FAS aids in various bioinformatics analyses by providing a detailed similarity score, which can be used to infer functional and evolutionary relationships between proteins. Researchers can leverage CANDy to investigate protein domain architectures and then utilize FAS to score protein structures, providing deeper insights into their functional and evolutionary relationships.

## 4. Conclusion

The properties of a protein are influenced by all of its domains. In enzymes, auxiliary domains play pivotal roles in substrate binding and they can serve as adaptable linkers that connect interacting domains. Identifying these domains within a given protein sequence of interest is a complex task that necessitates the use of specialized tools such as hidden Markov models (HMMs) and position-specific scoring matrices. CANDy leverages these resources provided by InterPro and incorporates multiple layers of analysis, enabling the automatic and accurate identification of protein domains for nearly all members of a CAZy family.

In conclusion, CANDy can assist studies involving the discovery, characterization, evolution and engineering of CAZymes in several ways. First, it provides a straightforward and clear overview of the diversity of domain architectures in an enzyme family. Second, it facilitates the selection of interesting candidates for further experimental studies. As demonstrated in family GH48, the characterized proteins certainly do not cover the entire diversity of domain architectures that exist in nature. Third, CANDy can significantly streamline the process of modular engineering of CAZymes. Its results enable the efficient extraction of subdomain sequences, and it provides information on whether particular domain architectures and connections are likely to yield a functional enzyme. This information may support the (semi-)rational design of novel chimaeras proteins. Finally, CANDy enables the exploration of phylogenetic relationships at both the sequence and domain levels and provides a visual depiction on potential gene fusion, fission and HGT events. This information proves invaluable when examining relationships between sequence, structure, and function or when pursuing protein engineering through ancestral sequence reconstruction.

## Supporting information

S1 Fig Raw phylogenetic tree as output from CANDy for family GH48. The leaf labels are annotated with their respective domain architecture, light blue bars: catalytic domain; black ellipse: CBM, green hexagon: dockerin, yellow hexagon: immunoglobulin-like domain; purple diamond: non-viral sialidases; orange triangle: oligoxyologlucan reducing end-specific cellobiohydrolase; pink triangle: glycoside hydrolase family 10, turquois left pointing pentagram: concanavalin A-like lectins/glucanases; dark pink right pointing pentagram: keratinocyte proline-rich protein; grey up pointing pentagram: selenoprotein W; black down pointing pentagram: clostridium cellulosome enzymes repeated domain signature; blue octagon: calX-like. Characterized proteins are colored, blue: EC 3.2.1.14; red: EC 3.2.1.176; green: EC 3.2.1.91; yellow: EC3.2.1.14.
(TIF)

S2 Fig Phylogenetic tree depicting the Actinomycetote (light green) and Insecta (light yellow) clades. The two groups of Actinomycetote proteins are marked with I and II. Local support values for each split are shown and were estimated using the Shimodaira-Hasegawa test and based on 1000 resamples. Other taxonomic groups including Mycoccota, Chloroflexota, Pseudomonadota and Bacteroidota are collapsed and represented by the grey triangle. The leaf labels are annotated with their respective domain architecture, dark blue bars: catalytic domain; yellow ellipse: CBM, light blue diamonds: immunoglobulin-like domain; green pentagon: selenoprotein W-like domain; purple hexagon: CalX-like domain; purple diamond: keratinocyte proline-rich protein-like domain. Characterized proteins are colored, yellow: EC 3.2.1.14; red: EC 3.2.1.176.
(TIF)

S1 Table Domain name curation. The results of the interactive domain name curation step. The left column represents the selected custom domain names. The right column contains the InterPro names that are included in each custom domain.
(PDF)

S2 Table Summary of all found domain architectures in family GH48. Taxonomy abbreviations: E: Eukaryotes; B: Bacteria; V: Viruses; U: Unknown.
(PDF)

S3 Table P-values after comparison of the mean percent identity between the catalytic domains of two groups Actinomycetota and Insecta, utilizing the Kruskal-Wallis test followed by Dunn’s post-hoc analysis. (PDF)

S4 Table P-values after comparison of the mean percent identity between the catalytic domains, CBMs and immunoglobulin-like domains of all Actinomycetota sequences included in the phylogenetic analysis, utilizing the Kruskal-Wallis test followed by Dunn’s post-hoc analysis. (PDF)

## Abbreviations

CANDy: Carbohydrate-active eNzyme Domain analysis tool
CAZy: Carbohydrate-active Enzyme database
CBM: carbohydrate Binding Module
GH: Glycoside Hydrolases
HGT: Horizontal Gene Transfer
