# Supplementary figures and images for: CANDy: Automated analysis of domain architectures in carbohydrate-active enzymes
Source: PLoS One. 2024 Jul 11;19(7):e0306410. doi: 10.1371/journal.pone.0306410 (PMC11238990; doi:10.1371/journal.pone.0306410)

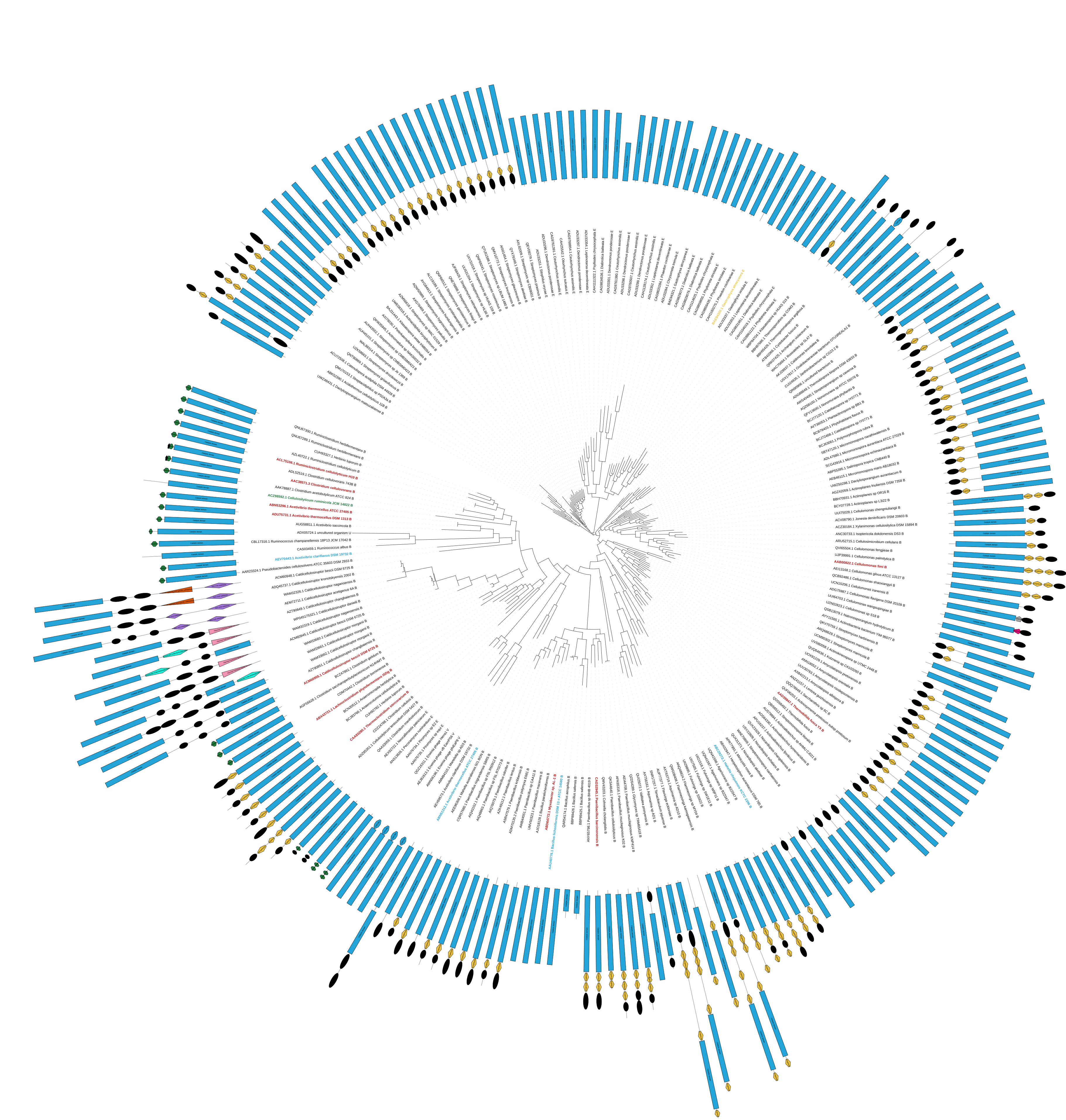

Supplement: S1 Fig — The leaf labels are annotated with their respective domain architecture, light blue bars: catalytic domain; black ellipse: CBM, green hexagon: dockerin, yellow hexagon: immunoglobulin-like domain; purple diamond: non-viral sialidases; orange triangle: oligoxyologlucan reducing end-specific cellobiohydrolase; pink triangle: glycoside hydrolase family 10, turquois left pointing pentagram: concanavalin A-like lectins/glucanases; dark pink right pointing pentagram: keratinocyte proline-rich protein; grey up pointing pentagram: selenoprotein W; black down pointing pentagram: clostridium cellulosome enzymes repeated domain signature; blue octagon: calX-like. Characterized proteins are colored, blue: EC 3.2.1.14; red: EC 3.2.1.176; green: EC 3.2.1.91; yellow: EC3.2.1.14. (TIF) [file pone.0306410.s001.tif]

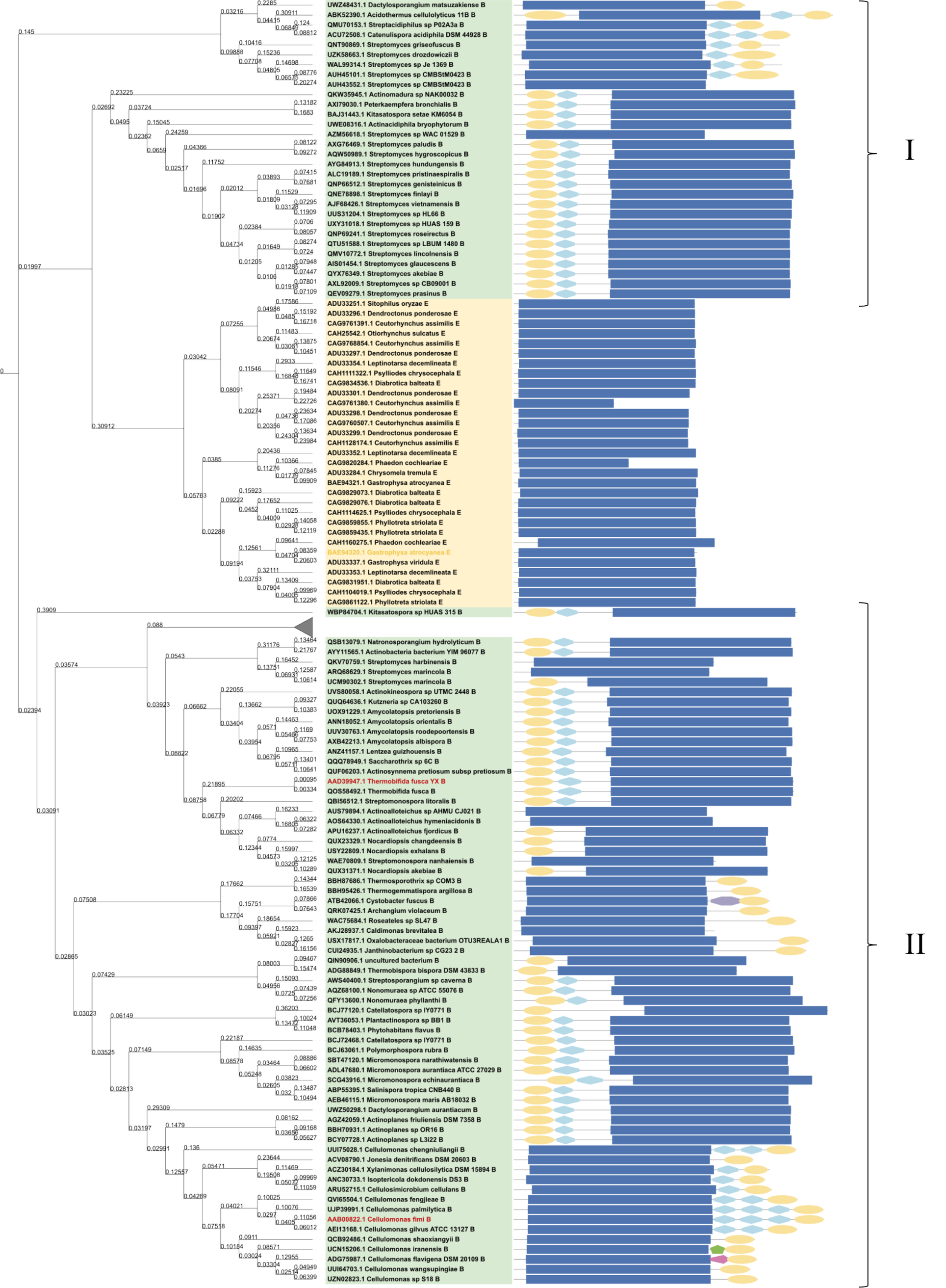

Supplement: S2 Fig — The two groups of Actinomycetote proteins are marked with I and II. Local support values for each split are shown and were estimated using the Shimodaira-Hasegawa test and based on 1000 resamples. Other taxonomic groups including Mycoccota, Chloroflexota, Pseudomonadota and Bacteroidota are collapsed and represented by the grey triangle. The leaf labels are annotated with their respective domain architecture, dark blue bars: catalytic domain; yellow ellipse: CBM, light blue diamonds: immunoglobulin-like domain; green pentagon: selenoprotein W-like domain; purple hexagon: CalX-like domain; purple diamond: keratinocyte proline-rich protein-like domain. Characterized proteins are colored, yellow: EC 3.2.1.14; red: EC 3.2.1.176. (TIF) [file pone.0306410.s002.tif]
