# Supplementary material for: CANDy: Automated analysis of domain architectures in carbohydrate-active enzymes
Source: PLoS One. 2024 Jul 11;19(7):e0306410. doi: 10.1371/journal.pone.0306410 (PMC11238990; doi:10.1371/journal.pone.0306410)
Supplement: S1 Table — The results of the interactive domain name curation step. The left column represents the selected custom domain names. The right column contains the InterPro names that are included in each custom domain. (PDF) [file pone.0306410.s003.pdf]

Table S1: Domain name curation. The results of the interactive domain name curation step. The left column represents the selected custom domain names. The right column contains the InterPro names that are included in each custom domain.

**Custom domain name**	**InterPro nomenclature**
Catalytic domain	Six-hairpin glycosidases<br>Glycosyl hydrolase family 48<br>(Trans)glycosidases
Immunoglobulin-like	Fibronectin type 3 domain<br>Bacterial Ig domain<br>E set domains, Secretion system C-terminal sorting domain<br>Fibronectin type III<br>Immunoglobulins<br>Repeats in polycystic kidney disease 1 (PKD1) and other proteins<br>PKD domain
Dockerin	Type I dockerin repeat domain<br>Cellulose docking domain, dockering<br>Type II dockerin repeat domain<br>Type I dockerin domain<br>Dockerin domain
CBM	Carbohydrate-binding domain<br>Cellulose binding domain<br>Carbohydrate-binding module 64<br>Cellulose or protein binding domain<br>CBM2 (Carbohydrate-binding type-2) domain profile
