# Supplementary material for: CANDy: Automated analysis of domain architectures in carbohydrate-active enzymes
Source: PLoS One. 2024 Jul 11;19(7):e0306410. doi: 10.1371/journal.pone.0306410 (PMC11238990; doi:10.1371/journal.pone.0306410)
Supplement: S2 Table — Taxonomy abbreviations: E: Eukaryotes; B: Bacteria; V: Viruses; U: Unknown. (PDF) [file pone.0306410.s004.pdf]

Table S1: Summary of all found domain architectures in family GH48. Taxonomy abbreviations: E: Eukaryotes; B: Bacteria; V: Viruses; U: Unknown.

**Predicted domain architecture**	**Predicted number of sequences included**	**Taxonomy**	**Example protein (GenBank ID)**	**Known specificity**
Catalytic domain + CBM + CBM	1	E	ANS13826.1	
Catalytic domain + Catalytic domain + CBM + CBM	1	B	AEV69573.1	
Catalytic domain + Dockerin	24	B, U	AEV70443.1†	3.2.1.4, 3.2.1.176
CBM + Catalytic domain + Immunoglobulin-like + CBM	4	B	ABK52390.1	
CBM + Immunoglobulin-like + Catalytic domain	287	B	AAD39947.1†	3.2.1.176
CBM + Catalytic domain	17	B	ADG88849.1	
Catalytic domain	156	B, E	AAU40776.1†	3.2.1.4, 3.2.1.14, 3.2.1.91, 3.2.1.176
Catalytic domain + Immunoglobulin-like + Immunoglobulin-like + Immunoglobulin-like + CBM	13	B	AAB00822.1†	3.2.1.176
Catalytic domain + Immunoglobulin-like + Immunoglobulin-like + CBM	18	B	ABC29272.1†	3.2.1.4, 3.2.1.176
Catalytic domain + Immunoglobulin-like + CBM	67	B	ABX43721.1†	3.2.1.176
Catalytic domain + CBM + Immunoglobulin-like + Immunoglobulin-like + Immunoglobulin-like + Immunoglobulin-like + Catalytic domain + Immunoglobulin-like	5	B	AXT56161.1‡	
Immunoglobulin-like + Catalytic domain + Immunoglobulin-like	1	B	AXT63719.1	
Catalytic domain + CBM	30	B	ABN51312.1†	3.2.1.4
Non-viral sialidases + Non-viral sialidases + CBM +	1	B	AEM72711.1‡	

CBM + CBM + Catalytic domain				
Non-viral sialidases + Oligoxyloglucan reducing end-specific cellobiohydrolase + CBM + CBM + Catalytic domain	3	B	ACM60948.1‡	
Glycosyl hydrolase family 10 + CBM + CBM + Catalytic domain	3	B	ACM60945.1‡	
Catalytic domain + CBM + CBM + CBM + Catalytic domain	7	B	ACM60955.1†	3.2.1.176
Glycosyl hydrolase family 10 + CBM + Concanavalin A-like lectins/glucanases + Catalytic domain	1	B	WP045175321.1‡	
Catalytic domain + CBM + CBM + CBM + CBM + Catalytic domain	1	B	WAM33662.1‡	
Concanavalin A-like lectins/glucanases + Catalytic domain + CBM + CBM + CBM + Catalytic domain	1	B	WAM33661.1‡	
Glycosyl hydrolase family 10 + CBM + CBM + CBM + Catalytic domain	1	B	WAM33660.1‡	
Non-viral sialidases + Non-viral sialidases + CBM + CBM + Catalytic domain	1	B	WAM32326.1‡	
Catalytic domain + CBM + CBM + Concanavalin A-like lectins/glucanases + Catalytic domain	1	B	WAM32319.1‡	
Catalytic domain + Keratinocyte proline-rich protein + CBM	1	B	ADG75987.1	
Catalytic domain + Selenoprotein W + CBM	1	B	UCN15206.1‡	
Catalytic domain + Dockerin + Clostridium cellulosome enzymes repeated domain signature	3	B	AAC38571.3†	3.2.1.176
Catalytic domain + Immunoglobulin-like + Immunoglobulin-like	1	B	QMV43203.1	
Catalytic domain + CalX-like + CBM	1	B	ATB42066.1	

CalX-like + Catalytic domain	7	B,V	QGZ16311.1
Catalytic domain + CBM + Immunoglobulin-like + Immunoglobulin-like + Immunoglobulin-like + Catalytic domain + Immunoglobulin-like	3	B	ANQ48824.2‡
Catalytic domain + Immunoglobulin-like	1	B	AUP77377.1
Catalytic domain + Immunoglobulin-like + Immunoglobulin-like + Immunoglobulin-like	1	B	UII29895.1‡
Catalytic domain + Immunoglobulin-like + Immunoglobulin-like + CBM + Immunoglobulin-like	2	B	UII27505.1‡
Catalytic domain + Dockerin + CBM	1	E	AEX92722.1
Catalytic domain + Immunoglobulin-like + Immunoglobulin-like + CBM + CBM	2	B	AFK65316.1
Catalytic domain + Dockerin + Dockerin	2	E	AAN76734.1

†Characterized protein

‡No AF model available on UniProt
