# Supplementary material for: CANDy: Automated analysis of domain architectures in carbohydrate-active enzymes
Source: PLoS One. 2024 Jul 11;19(7):e0306410. doi: 10.1371/journal.pone.0306410 (PMC11238990; doi:10.1371/journal.pone.0306410)
Supplement: S3 Table — (PDF) [file pone.0306410.s005.pdf]

Table S1: p-values after comparison of the mean percent identity between the catalytic domains of two groups Actinomycetota and Insecta, utilizing the Kruskal-Wallis test followed by Dunn's post-hoc analysis.

| | **Actinomycetota (I)** | **Actinomycetota (II)** | **Insecta** |
|---|---|---|---|
| **Actinomycetota (I)** | 1.000000E+00 | 1.507972E-11 | 1.595730E-34 |
| **Actinomycetota (II)** | 1.507972E-11 | 1.000000E+00 | 1.654714E-05 |
| **Insecta** | 1.595730E-34 | 1.654714E-05 | 1.000000E+00 |
