# Supplementary material for: CANDy: Automated analysis of domain architectures in carbohydrate-active enzymes
Source: PLoS One. 2024 Jul 11;19(7):e0306410. doi: 10.1371/journal.pone.0306410 (PMC11238990; doi:10.1371/journal.pone.0306410)
Supplement: S4 Table — (PDF) [file pone.0306410.s006.pdf]

Table S1: p-values after comparison of the mean percent identity between the catalytic domains, CBMs and immunoglobulin-like domains of all Actinomycetota sequences included in the phylogenetic analysis, utilizing the Kruskal-Wallis test followed by Dunn's post-hoc analysis.

	**Catalytic domain**	**CBM**	**Immunoglobulin-like**
**Catalytic domain**	1.000000E+00	2.595453E-204	2.068178E-135
**CBM**	2.595453E-204	1.000000E+00	5.158254E-02
**Immunoglobulin-like**	2.068178E-135	5.158254E-02	1.000000E+00
